# Novel Risk Associations between microRNA Polymorphisms and Gastric Cancer in a Chilean Population

**DOI:** 10.3390/ijms23010467

**Published:** 2021-12-31

**Authors:** Natalia Landeros, Alejandro H. Corvalan, Maher Musleh, Luis A. Quiñones, Nelson M. Varela, Patricio Gonzalez-Hormazabal

**Affiliations:** 1 Advanced Center for Chronic Diseases, Pontificia Universidad Católica de Chile, Santiago 8330034, Chile; natalialanderos@udec.cl (N.L.); acorvalan@uc.cl (A.H.C.); 2 Department of Hematology-Oncology, School of Medicine, Pontificia Universidad Católica de Chile, Santiago 8330034, Chile; 3 Department of Surgery, University of Chile Clinical Hospital, Santiago 8380456, Chile; mmusleh@hcuch.cl; 4 Department of Basic-Clinical Oncology, School of Medicine, University of Chile, Santiago 8380453, Chile; lquinone@uchile.cl (L.A.Q.); varelanel@gmail.com (N.M.V.); 5 Latin American Network for the Implementation and Validation of Pharmacogenomic Clinical Guidelines (RELIVAF-CYTED), 28015 Madrid, Spain; 6 Human Genetics Program, Institute of Biomedical Sciences (ICBM), School of Medicine, Universidad de Chile, Santiago 8380453, Chile

**Keywords:** gastric cancer, miRNA, polymorphism, *Helicobacter pylori*, *cag* pathogenicity island

## Abstract

Gastric cancer (GC) is the fifth leading cause of cancer deaths in the world, with variations across geographical regions and ethnicities. Emerging evidence indicates that miRNA expression is dysregulated in GC and its polymorphisms may contribute to these variations, which has yet to be explored in Latin American populations. In a case-control study of 310 GC patients and 311 healthy donors from Chile, we assessed the association of 279 polymorphisms in 242 miRNA genes. Two novel polymorphisms were found to be associated with GC: rs4822739:C>G (miR-548j) and rs701213:T>C (miR-4427). Additionally, rs1553867776:T>TCCCCA (miR-4274) and rs12416605:C>T (miR-938) were associated with intestinal-type GC, and rs4822739:C>G (miR-548j) and rs1439619:T>G (miR-3175) with TNM I-II stage. The polymorphisms rs6149511:T> TGAAGGGCTCCA (miR-6891), rs404337:G>A (miR-8084), and rs1439619:T>G (miR-3175) were identified among *H.pylori*-infected GC patients and rs7500280:T>C (miR-4719) and rs1439619:T>G (miR-3175) were found among *H. pylori cag*PAI+ infected GC cases. Prediction analysis suggests that seven polymorphisms could alter the secondary structure of the miRNA, and the other one is located in the seed region of miR-938. Targets of miRNAs are enriched in GC pathways, suggesting a possible biological effect. In this study, we identified seven novel associations and replicated one previously described in Caucasian population. These findings contribute to the understanding of miRNA genetic polymorphisms in the GC pathogenesis.

## 1. Introduction

Gastric cancer (GC) is the fifth leading cause of cancer deaths in the world [1] with variations across geographical regions as well as ethnicities [2,3]. The highest GC mortality rates are found in Asia, Eastern Europe and Latin America and are mostly related to the advanced stage of disease [4,5]. The combination of heritage, environmental factors, diet, and infectious agents (i.e., *Helicobacter pylori* and Epstein-Barr virus) contribute to geographical and ethnic variations resulting in two main types of GC, intestinal and diffuse-type [6]. The former is preceded by sequentially ordered precancerous histological changes and is etiologically associated with environmental exposures and the chronic infection of *H. pylori*, principally strains harboring the *cag* pathogenicity island (*cag*PAI) [7]. In contrast, the latter arises “de novo” and has host-genetic architecture which plays a major role in its development [8,9].

MicroRNAs (miRNAs) are single-stranded noncoding RNAs between 18 and 24 nucleotides in length and evolutionarily conserved [10,11]. Genetic polymorphisms in miRNAs may alter the transcription of the pri-miRNA and the processing of pre-miRNA through modifying the base pairing in the secondary structure. If the genetic polymorphism is found in the seed region, it could affect miRNA:mRNA interaction without necessarily affecting the level of miRNA [12]. There is some evidence that genetic variations in miRNA genes have clinical relevance [13].

In GC, several miRNAs have been reported to be differentially expressed between tumor and normal tissues [14]. In addition, various research groups have been analyzing the role of genetic polymorphisms of miRNAs in GC under the rationale of hypothesis-driven (candidate gene) approach [15]. These studies, mostly conducted in Asia, evidenced the role of miRNA and its polymorphisms as potential new targets for diagnosis and treatment of GC [16,17,18,19,20,21,22,23]. Unfortunately, in the Latin American population, in particular in the Chilean population which has one of the highest GC mortality rates [1,2,3], no studies have been conducted in this area.

Therefore, we analyzed all genetic polymorphisms of miRNAs deposited in miRBase, including those which have already been evaluated in other studies [16,17,18,19,20,21,22,23]. Here, we identified seven previously unreported genetic polymorphisms: rs4822739 in miR-548j, rs701213 in miR-4427, rs15538677 in miR-4274, rs12416605 in miR-938, rs1439619 in miR-3175, rs61449511 in miR-6891, rs404337 in miR-8084, and rs7500280 in miR-4719. In silico analysis predicted changes in transcription, processing and binding through modifications in the secondary structure which suggests a functional effect in GC related pathways.

## 2. Results

**Figure 1:**
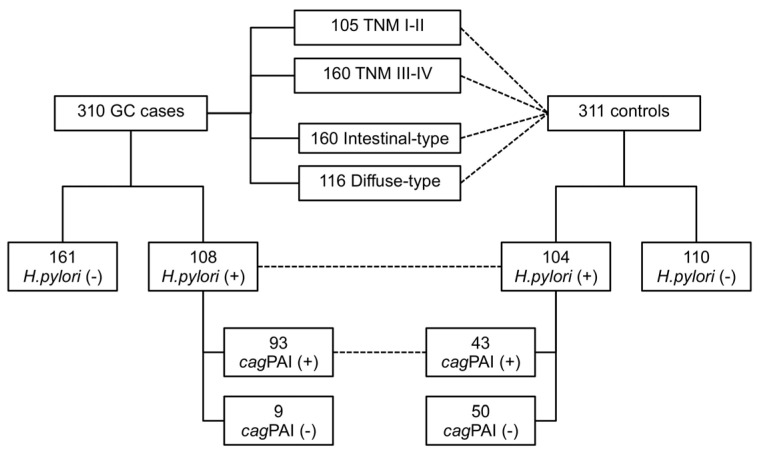
Flowchart of the comparisons among subsets. Dashed lines represent the stratified analysis.

To evaluate the clinical significance of miRNA polymorphisms, 310 GC cases and 311 controls were analyzed by Lauren’s histological classification, TNM stage, and *H. pylori* infection status, including the presence of *cag*PAI (Figure 1, Table 1). A subset of 269 (86.8%) cases and 214 (68.8%) controls were analyzed for *H. pylori* infection status and the presence of *cag*PAI by real-time PCR. One hundred and eight GC cases (40.1%) and 104 controls (48.6%) resulted positive for *H. pylori* infection. Of these, 93 GC and 43 controls were infected with *cag*PAI-positive strains. Information regarding the TNM stage was available for 265 GC cases, where 105 were in the TNM I-II stage (39.6%) and 160 were in the TNM III-IV stage (60.4%).

### 2.1. Polymorphisms in miRNAs Associated with Gastric Cancer

Of the 1,772 miRNAs available in miRbase (release 22.1) [11], 279 genetic polymorphisms were identified in 242 miRNA genes (Table S1, see Supplementary Materials). An association analysis identified eight polymorphisms (rs701213, rs4822739, rs12416605, rs1553867776, rs14396919, rs6149511, rs404337, and rs7500280) (Table 2) of which seven resulted in novel GC associations, all except for rs12416605 [24]. The eight associated polymorphisms have a R-squared value > 0.3, which means that have a sufficient quality of imputation. The polymorphisms rs701213 in miR-4427 and rs4822739 in miR-548j were associated with reduced risk and susceptibility to GC, respectively. Two polymorphisms were found to be associated with the intestinal-type of GC, the decreased risk variant rs12416605 in miR-938 and susceptibility variant rs1553867776 in miR-4274. No miRNA polymorphisms were associated with the diffuse type of GC. A stratified analysis according to the TNM stage found a susceptibility association between stages TNM I-II and the polymorphism rs4822739 in miR-548j. However, the polymorphism rs1439619 in miR-3175 was found to be a reduced risk variant in the same group of tumors. No miRNA polymorphisms were associated with patients at TNM III-IV stages (Table 2). To assess if these novel genetic polymorphisms are associated with GC in *H. pylori*-infected patients, we performed an association analysis in a subgroup of 108 cases and 104 controls. In the GC cases we found a decreased risk association with the polymorphism rs1439619 in miR-3175 but a susceptibility association with polymorphisms rs6149511 in miR-6891 and rs404337 in miR-8084 (Table 2). To further assess the influence of the oncogenic strain *cag*PAI of *H. pylori*, we carried out an association analysis in 93 *cag*PAI-positive cases and 43 *cag*PAI-positive controls. This substudy identified an inverse association with risk of GC in the case of rs1439619 in miR-3175 and a susceptibility association in the case of rs7500280 in miR-4719. Of note, the protective effect of the rs1439619 polymorphism was found in both *H. pylori* and *cag*PAI-positive strains.

### 2.2. Prediction of the Effect of Polymorphisms on the Secondary Structure of pre-miRNAs

**Figure 2:**
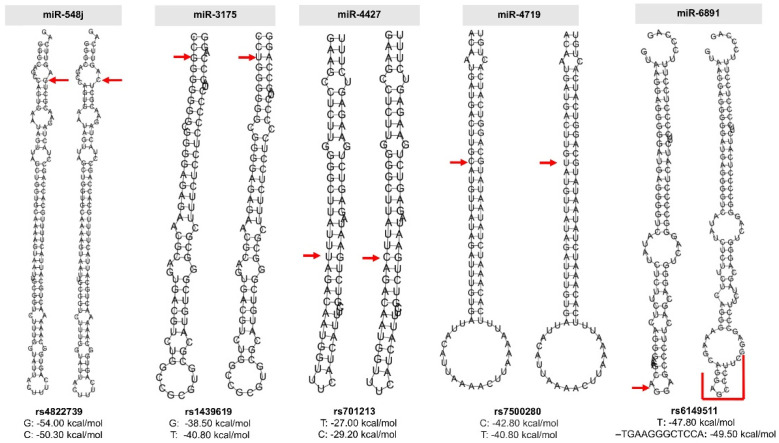
Secondary structure modeling of genetic variants in the pre-miRNAs. The predicted structures of pre-miRNAs with common allele (left) and the variant (right) are shown. A red arrow indicates the position of the allele. The free energies of both alleles are also shown.

Of the eight miRNA polymorphisms with significant associations, five were located in the pre-miRNAs of their respective miRNA genes (rs4822739, rs1439619, rs701213, rs7500280, and rs6149511). To assess the possible effect of these polymorphisms, we modeled both alleles of each of the five miRNAs sequences, in silico. The modification of the minimum free energy of these structures (ΔΔG) was used to evaluate the stability of the pre-miRNAs as described. The rs4822739 polymorphism at position 105 in the pre-miRNA-548j and corresponds to a G>C substitution. Our in silico analysis predicted free energy changes from −54.0 kcal/mol for nucleotide G in the transcribed pre-miRNA to −50.3 kcal/mol for the nucleotide C (ΔΔG 3.7 kcal/mol). In addition, the nucleotide C abolishes a C:G base pairing in an internal loop (Figure 2). The rs1439619 polymorphism corresponds to a T>G substitution at position 3 of pre-miR-3175. This change modifies the secondary structure of the pre-miRNA by eliminating an internal loop, decreasing the energy of the structure (ΔΔG 2.3 kcal/mol), and possibly making it a more unstable transcript (Figure 2). The substitution could alter the affinity with which Drosha binds to process the pre-miRNA. Another polymorphism is rs701213, located at position 21 in the pre-miR-4427 and corresponds to a T>C substitution. This substitution modifies the U:G non-Watson—Crick base-pairing to a more stable C:G pairing. The free energy changed from −27.0 kcal/mol (nucleotide C) to −29.2 kcal/mol (nucleotide T) (ΔΔG −2.2 kcal/mol), increasing the stability of the structure. In miR-4719 the rs7500280 polymorphism changes the U:G non-Watson—Crick base-pairing to C:G pairing. In terms of energy, the structure is more stable, which could alter the efficiency of Drosha processing when the risk allele is present (Figure 2). In pre-miR-6891, the rs6149511 polymorphism corresponds to an insertion of TGAAGGGCTCCA in position 44 which changes energy from −47.80 kcal/mol in the ancestral allele to −49.50 kcal/mol in the insertion allele, significantly altering the loop terminal region (Figure 2). Taken together, the structural alterations in pre-miRNAs suggest that rs4822739 in miR-548j, rs1439619 in miR-3175, rs701213 in miR-4427, rs7500280 in miR-4719, and rs6149511 in miR-6891 could alter the levels of mature miRNAs.

### 2.3. Prediction of the Effect of the rs404337 Polymorphism on the Secondary Structure of the Mature miR-8084

**Figure 3:**
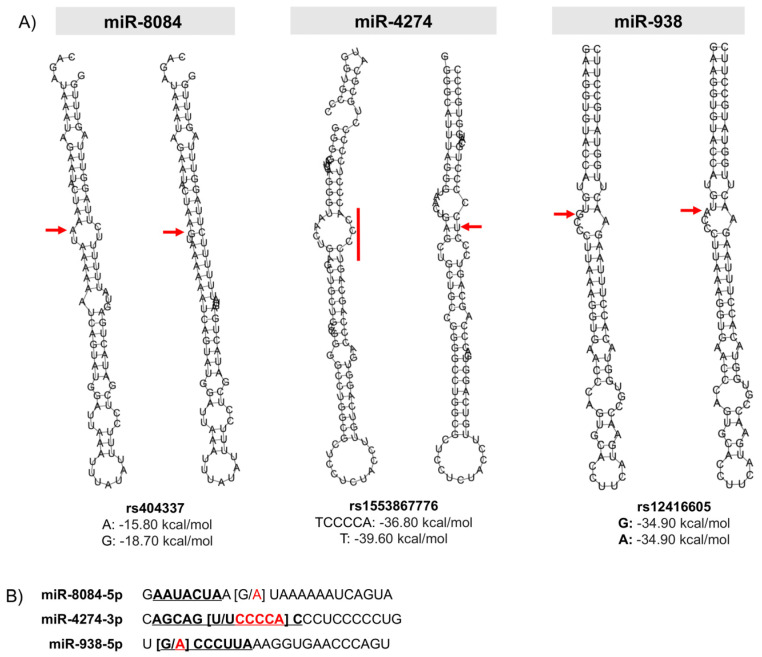
Secondary structure modeling of genetic variants in mature miRNAs. (**A**) The predicted structures of pre-miRNAs with common allele (left) and the variant (right) are shown. A red arrow indicates the position of the allele. The free energies of both alleles are also shown. (**B**) The sequence of the mature miRNA is shown in bold, and the seed region is underlined.

The rs404337 polymorphism corresponds to a G>A substitution and is located at position 10 of the mature miR-8084-5p, outside the seed region. This substitution generates a change of free energy in the secondary structure from −18.7 kcal/mol in the nucleotide G to −15.80 kcal/mol in the nucleotide A (ΔΔG 2.9 kcal/mol), creating two new internal loops and making the structure more unstable (Figure 3A). Furthermore, this polymorphism is two nucleotides out from the seed region, so it has the potential to modify the interaction between this miRNA and its target mRNAs (Figure 3B).

### 2.4. Prediction of the Effect on the Secondary Structure of rs1553867776 and rs12416605 Polymorphisms in the Seed Region of miRs-4274-3p and -938

The rs1553867776 polymorphism corresponds to the insertion of six nucleotides (TCCCCA) in the last nucleotide of the seed region of mature miR-4274-3p (Figure 3B). This insertion instigates an important structural change, causing an energy change of ΔΔG 2.8 kcal/mol. Therefore, this polymorphism could alter the processing of Drosha and Dicer (Figure 3A). The rs12416605 polymorphism is located in the seed region of miR-938 and changes the first nucleotide of the region but does not modify the secondary structure.

### 2.5. Predicted Targets of Novel miRNA Genes Containing Polymorphisms in Gastric Cancer

**Figure 4:**
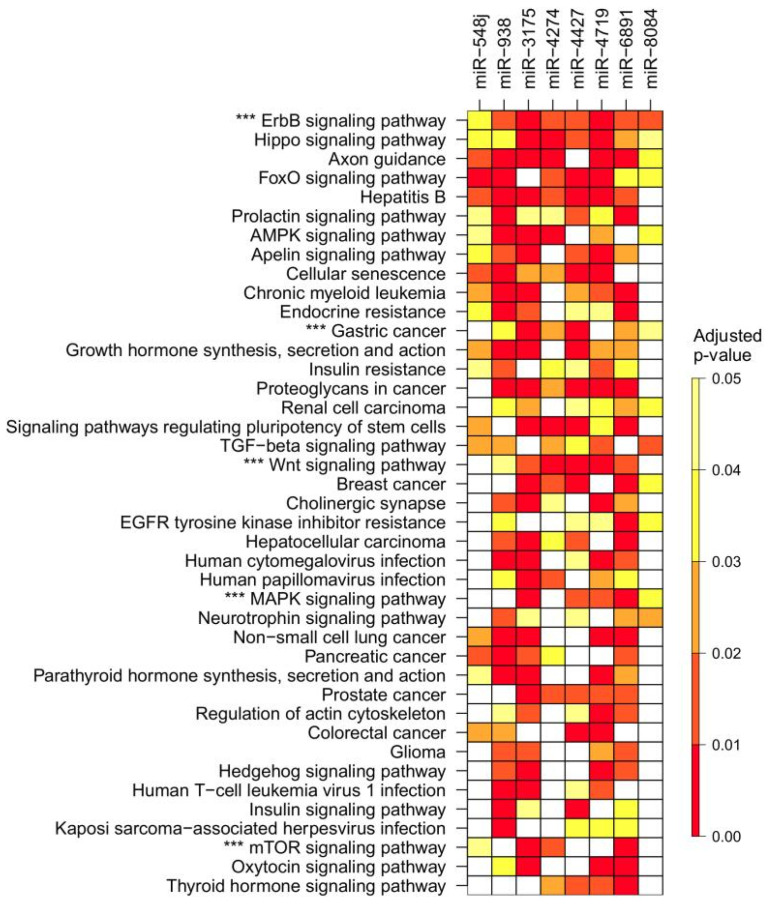
A heatmap of the most enriched pathways of the eight miRNAs analyzed is shown. A total of 41 pathways were targeted by at least half (50%) of the studied miRNAs. Remarkably, the “gastric cancer” pathway was targeted by six miRNAs. This pathway involved other displayed pathways (***) demonstrating the redundancy of these findings. Adjusted *p*-value after Bonferroni correction for multiple comparisons.

We analyzed whether the eight novel miRNA genes containing polymorphisms regulate pathways associated with GC pathogenesis using an enrichment analysis of the Kyoto Encyclopedia of Genes and Genomes (KEGG) pathways. The predicted pathways for each miRNA are presented in Figure S1. A consolidated heatmap shows pathways that are simultaneously targeted by four or more miRNAs (Figure 4). Interestingly, the “gastric cancer” pathway (map 05226) was enriched in six out of the eight miRNAs and according to KEGG, this pathway includes other highly predicted pathways, confirming the redundancy of our findings.

## 3. Discussion

The contribution of miRNA genetic polymorphisms to GC is poorly understood outside Asian countries. In particular, this contribution has not been well-documented in Latin America, the region with the third-highest GC mortality rates in the world [1]. In this investigation, eight genetic polymorphisms of the 279 listed in miRbase (release 22.1) [11] were found to be associated with GC in Chilean population. Among these polymorphisms, rs4822739, rs701213, rs12416605, rs1553867776, rs14396919, rs6149511, and rs404337 have not been previously reported. Two polymorphisms—rs4822739 in miR-548j and rs1553867776 in miR-4274—are of particular interest to this study, as they were associated with susceptibility to GC at early stages (TNM I-II) and to intestinal-type of GC, respectively. Conversely, polymorphisms rs701213 in miR-4427 and rs1439619 in miR-3175 were associated with a reduced risk of GC, particularly at the early stages in the case of rs1439619. Polymorphisms rs6149511 in miR-6891 and rs404337 in miR-8084 were found to be associated with susceptibility to GC in *H. pylori*-infected patients and polymorphism rs7500280 in miR-4719 with infection of its oncogenic strain *cag*PAI. Polymorphism rs1439619 in miR-3175 was associated with an overall reduced risk of developing GC at early stages as well as reduced risk in cases infected with *H. pylori*, particularly those with *cag*PAI oncogenic strain. 

In this study, we replicated the results described by Torruela-Loran et al. [24] in which the polymorphism rs12416605 in miR-938 was reported to be associated with GC in the Caucasian population. Of note, these authors found it in association with diffuse-type of GC whereas our findings linked it to the intestinal-type in Chilean population. However, further analysis was precluded by the number of unspecified histological subtypes of GC in the case of Torruela-Loran et al. [24]. The substitution of C or T alleles in this polymorphism showed that the former is expressed 1.49-fold more than the latter allele, supporting the association of this polymorphism with GC. The reduced risk of rs14396919 in miR-3175 is according to Kim et al. [25] who identified miR-3175 as a member of a miRNA signature associated with lymph node metastasis in GC at early stages (intramucosal stage). In other tumors such glioma and prostate, miR-3175 promotes cell proliferation, invasion and apoptosis [26,27]. Our ΔΔG analysis suggests that the substitution of T>G alleles modifies the secondary structure of the pre-miRNA expressing a more unstable transcript which might preclude protection against *H. pylori* infection and its oncogenic strain *cag*PAI. 

It has been reported that deregulation and variants of miRNA are associated with TNM and aggressiveness of cancer progression [28,29,30]. In the present study, we show that rs4822739 (miR-548j) is associated with GC, in particular among patients with TNM I-II stage. On the other side, rs404337 (miR-8084) confers risk to GC in *H. pylori*-infected individuals. No studies have been published on the relationship of these miRNAs with GC. Evidence is scarce for the functionality of miR-548j and miR-8084 in other cancers. The former promotes invasion and metastasis in breast cancer and the latter is upregulated in breast cancer tumors and enhances migration and invasion by inducing epithelial-to-mesenchymal transition [31,32]. To date, no studies have been published describing the role of miR-4427, miR-6891, miR-4274, and miR-4719 in other tumors. Our prediction analysis suggests that the polymorphisms described in this study could be functional, either by changing the levels of their miRNAs or through interacting with their target genes. Therefore, these miRNA polymorphisms have the potential to affect a plethora of cellular processes in which they participate. It is noteworthy that the KEGG pathway enrichment analysis revealed that target genes associated with the described miRNAs belong to the “gastric cancer” pathway. Since an individual miRNA can target several genes within the same cellular pathway, our analysis confirms that other oncogenic pathways were included in the “gastric cancer” pathway, such as ErbB, TGF-β, Wnt, MAPK, and mTOR pathways. Of note, the “Epithelial cell signaling in *Helicobater pylori* infection” pathway was enriched for miR-4719 which was associated with GC in subjects infected with *cag*PAI-positive strains. The most commonly studied miRNA genetic polymorphisms were also evaluated in this study: rs11614913 in miR-196a2, rs2910164 in miR-146a, and rs2292832 in miR-149 [17,18,19]. Although other polymorphisms were initially associated with GC (rs4938723 in miR-34b/c, rs895819 in miR-27a, rs3746444 in miR-499, rs2043556 in miR-605, and rs6505162 in miR-423), current meta-analyses have not confirmed these associations [17,20,21,22,23]. Remarkably, none of these polymorphisms were found to be associated with GC in the present study (Table S2).

The present study has some limitations. It does not necessarily represent the general population of Latin America since the study is restricted to Chile, which has a limited contribution to the Caucasian and Amerindian components. The sample size is not large, though it was sufficient to reach the desired statistical power (β > 0.80) for all the associated polymorphisms. Data regarding environmental risk factors were not available, which prevented the assessment of gene–environment interactions. Finally, the target analysis was restricted to Targetscan and miRmap, since the other platforms were not updated to the last release of miRbase at the time of access (release 22.1) [11].

In conclusion, in Latin America, a region with a high GC mortality rate, the contribution of miRNA genetic polymorphisms is poorly understood. In this scenario, we interrogated 279 genetic polymorphisms and identified seven unreported associations. In addition, we identified a previously described association of rs12416605 with GC in Caucasian population. Our in silico analysis of the structure stability and predicted targets related with cancer pathways suggest a functional effect for these polymorphisms. Further in vitro as well as translational studies will be needed to validate and extend to a clinical setting the results obtained in this investigation.

## 4. Materials and Methods

### 4.1. Subjects

Three hundred ten individuals (202 men and 108 women) with a mean age of 64.1 years (standard deviation 12.1 years, range 25 years to 93 years) were recruited at the time of surgical resection between 2001 and 2018 from Hospital Clínico Universidad de Chile and Biobanco de Tejidos y Fluidos de la Universidad de Chile (BTUCH), Hospital del Salvador, Hospital Barros Luco Trudeau, Hospital San Juan de Dios and Hospital Militar de Santiago at Santiago Metropolitan Region, Chile. All patients had confirmed the histopathological diagnosis of gastric adenocarcinoma. TNM stage was obtained from the histopathological report. The control group corresponds to 311 individuals (187 men and 124 women, mean age 51.1 years, standard deviation 15.6 years, ranging from 18 to 82) with no personal history of cancer. Blood samples were collected in EDTA vacutainers for all participants. A sample of gastric mucosa was taken from 269 cases and 224 controls. For cases, a fresh sample of gastric mucosa was obtained from the corpus distant to the tumor. In the case of controls, an upper gastrointestinal endoscopy was performed to rule out the presence of GC as well to take a fresh piece of gastric mucosa for *H. pylori* detection; none of them received previously therapy to eradicate *H. pylori*. This study was approved by the institutional review board of the University of Chile School of Medicine (#045/2015) in accordance with the Declaration of Helsinki. All participants signed informed consent to participate.

### 4.2. Genotyping

Genomic DNA was obtained from blood samples using the salting out and Proteinase K method, or according to the protocol described by Chomczynski and Sacchi [33]. The DNA was further purified using Monarch PCR and DNA cleanup columns (New England Biolabs, Ipswich, MA, USA). The Infinium Global Screening Array-24 BeadChip GSA-MD Version 1 (Illumina, San Diego, CA, USA) was used to genotype the DNA samples at the Human Genomics Facility (HuGe-F) in Erasmus MC, (Rotterdam, The Netherlands) according to the manufacturer’s protocol.

### 4.3. Genotype Imputation

Imputation was performed to obtain the genotype of polymorphisms not directly genotyped in the GSA array. The procedure is comprised of the following steps: quality control (QC) of genotyped polymorphisms, phasing of alleles, and imputation. QC of the genotyping data was performed according to the guidelines in Anderson et al. [34] using plink 1.9 (Available online: www.cog-genomics.org/plink/1.9/ (accessed on 5 March 2019)) [35]. After QC, the resulting *.bed file was converted to a *.vcf file using plink 1.9 --recode vcf command keeping autosomal polymorphisms with minor allele frequency (MAF) > 0.01. It resulted in a *.vcf file containing genotypes of 449,097 polymorphisms that passed filters and QC, and was submitted to SHAPEIT4 [36] with by-default parameters to phasing the alleles. Maps for autosomes were downloaded from https://github.com/odelaneau/shapeit4/tree/master/ maps (accessed on 20 November 2020). Imputation was performed with Minimac4 (Available online: https://github.com/statgen/Minimac4 (accessed on 28 October 2020)) using by-default parameters including—ignore duplicates and --cpu 8. The reference panel was based on 1000 Genomes Phase 3 (version 5) global (2504 samples) with parameter estimates in *.M3VCF file format downloaded from ftp://share.sph.umich.edu/minimac3/G1K_P3_M3VCF_FILES_WITH_ESTIMATES.tar.gz (accessed on 20 November 2020). Imputed genotypes with R^2^ < 0.3 were ignored in further analyses.

### 4.4. Association Analyses of Imputed Genotypes

The resulting *.dose.vcf.gz file from Minimac4 was converted to plink format (*.plink.dosage.gz) using DosageConvertor (Available online: https://github.com/Santy-8128/ DosageConvertor (accessed on 28 October 2020)) with by-default parameters. The genomic coordinates of 1772 pre-miRNA genes were obtained from miRBase (Available online: ftp://mirbase.org/pub/mirbase/CURRENT/genomes/hsa.gff3 (accessed on 18 December 2020), representing all the registered autosomal miRNA genes. The file was converted to *.bed using gff2bed from BEDOPS 2.4.26 (Linux sudo apt-get install bedops) and then to GRCh 37 assembly using Ensembl Assembly Converter (Available online: https://grch37.ensembl.org/Homo_sapiens/Tools/AssemblyConverter (accessed on 18 December 2020)). The *.plink.dosage.gz file in conjunction with the genomic coordinates of studied miRNA genes was used for the association study in plink1.9 with the --dosage command under the additive model. To adjust for population stratification, we obtained Principal component 1 (PC1) and PC2 using a set of set of 184,909 autosome polymorphisms obtained from Infinium Global Screening Array (Illumina) excluding—pruning-SNPs from extended regions of LD (r^2^ > 0.2) using plink 1.9—indep-pairwise. A nominal *p*-value < 0.01 was considered statistically significant for the adjusted model.

### 4.5. Stratified Analyses

Association analyses were performed in the following strata: diffuse histological type, intestinal histological type, TNM I and II, TNM III and IV, *H. pylori*-infected subject, and *H. pylori cag*PAI-positive infected patients. *H. pylori* colonization was determined by real time PCR in gastric mucosa DNA from available samples according to Kobayashi [37]. Samples which did not amplify the 16S gene were classified as *H. pylori*-negative. The status of *cag*PAI-positive or -negative was determined by amplification of the *cag*E gene by means of real time PCR as described [38].

### 4.6. Prediction of the Biological Effect of Associated Variants

RNAfold (Available online: http://rna.tbi.univie.ac.at/ (accessed on 10 May 2021)) was used to model the secondary structure of the pre-miRNAs and to calculate the minimum free energy (MFE) with both alleles. TargetScan Human 7.2 (retrieved using hoardeR 0.9.4-2 package for R cran) and miRmap v1.1 [39] were combined to predict the targets of each miRNA and to reduce false positives. The enrichment of those targets was used by the enrichKEGG function of clusterProfiler 3.18.0 R package [40]. The results of the enrichment were visualized using dotplot function (enrichplot 1.10.1 R package) and plot.matrix 1.5.2 R package.

## Figures and Tables

**Table 1:** Clinicopathological characteristics of gastric cancer cases and controls.

Variable	Gastric Cancer	Controls	*p*-Value
Female	108 (34.8%)	124 (39.9%)	
Male	202 (65.2%)	187 (60.1%)	0.225 ^1^
Age (SD)	64.1 (12.1)	51.1 (15.6)	<0.001 ^2^
Lauren’s classification			
Intestinal	160 (51.6%)	-	
Diffuse	116 (37.4%)	-	
Mixed	31 (10.0%)	-	
Not available	3 (1.0%)	-	
TNM			
I	55 (17.7%)	-	
II	50 (16.1%)	-	
III	148 (47.7%)	-	
IV	12 (3.9%)	-	
Not available	45 (14.5%)	-	
*H.pylori* status			
Positive	108 (40.1%)	104 (48.6%)	
Negative	161 (59.9%)	110 (51.4%)	0.077 ^1^
*cag*PAI status			
Positive	93 (86.1%)	43 (41.3%)	
Negative	9 (8.3%)	50 (48.1%)	<0.001 ^1^
Not available	6 (5.6%)	11 (10.6%)	

^1^ Chi-squared test, ^2^ *t*-test, SD: Standard deviation, TNM: Tumor, lymph Node, Metastasis cancer staging system, *cag*PAI: *cag* pathogenicity island

**Table 2:** Genetic polymorphisms associated with gastric cancer with the smallest p-value by allele model.

rsID (Gene Name)	Tested Allele	R^2^	Allelic Frequency (Cases/Controls)	AMR	OR ^1^	*p*-Value ^1^	OR ^2^	*p*-Value ^2^	OR ^3^	*p*-Value ^3^
All Gastric Cancer cases										
rs701213 T>C (miR-4427)	C	0.44	0.367/0.412	0.39	0.65	0.009	0.71	0.067	0.68	0.0212
rs4822739 C>G (miR-548j)	G	0.95	0.156/0.101	0.11	1.60	0.009	1.87	0.002	1.54	0.0153
Intestinal-type Gastric Cancer										
rs12416605 C>T(miR-938)	T	0.96	0.202/0.279	0.22	0.64	0.008	0.68	0.041	0.65	0.0109
rs1553867776 T>TCCCCA (miR-4274)	TCCCCA	0.79	0.91/0.855	0.90	2.08	0.005	1.95	0.018	1.98	0.0083
TNM I-II stage										
rs1439619 T>G (miR-3175)	G	0.91	0.520/0.623	0.52	0.59	0.002	0.62	0.014	0.63	0.0059
rs4822739 C>G (miR-548j)	G	0.95	0.179/0.111	0.11	1.90	0.006	1.98	0.008	1.81	0.0094
*H. pylori*-infected subjects										
rs1439619 T>G (miR-3175)	G	0.91	0.558/0.672	0.52	0.52	0.006	0.52	0.015	0.61	0.0229
rs6149511 T>TGAAGGGCTCCA(miR-6891)	TGAAGGGCTCCA	0.67	0.461/0.353	0.48	2.02	0.008	2.17	0.012	1.86	0.0130
rs404337 G>A (miR-8084)	A	0.80	0.888/0.820	0.83	2.56	0.009	2.91	0.009	1.97	0.0415
*H. pylori cag*PAI-positive subjects										
rs1439619 T>G (miR-3175)	G	0.91	0.538/0.688	0.52	0.44	0.009	0.48	0.034	0.52	0.0204
rs7500280 T>C (miR-4719)	C	0.80	0.569/0.406	0.59	2.25	0.009	2.56	0.011	2.25	0.0074

^1^ Adjusted for sex, principal component PC1 and PC2. ^2^ Adjusted for age, sex, PC1 and PC2. ^3^ Crude. AMR: Allele frequency in Ad Mixed Americans in 1000 genomes project, R^2^: R-squared (a metric of quality of imputation), OR: Odds ratio, *p*-value < 0.01 is considered significant.

## Data Availability

Data available on request due to ethical restrictions.

## Supplementary Materials

The following are available online at https://www.mdpi.com/article/10.3390/ijms23010467/s1.
