# Supplementary material for: Novel Risk Associations between microRNA Polymorphisms and Gastric Cancer in a Chilean Population
Source: Int J Mol Sci. 2021 Dec 31;23(1):467. doi: 10.3390/ijms23010467 (PMC8745138; doi:10.3390/ijms23010467)
Supplement: Supplementary file 1 [file ijms-23-00467-s001.zip › Table_S2.pdf]

Table S2. Association analysis of rs4938723 T>C (miR-34b/c), rs895819 T>C (miR-27a), rs3746444 A>G (miR-499), rs2043556 T>C (miR-605) and rs6505162 A>C (miR-423) with gastric cancer by allele model.

rsID (Gene name)	Tested allele	R^2	Allelic frequency (cases/controls)	AMR	OR[1]	p-value[1]	OR[2]	p-value[2]	OR[3]	p-value[3]
All Gastric Cancer cases										
rs4938723 T>C (miR-34b/c)	C		0.25/0.26	0.22	0.96	0.74	0.94	0.69	0.94	0.63
rs895819 T>C (miR-27a)	C		0.20/0.23	0.38	0.78	0.13	0.77	0.15	0.79	0.15
rs3746444 A>G (miR-499)	G		0.15/0.15	0.13	1.04	0.80	1.03	0.89	1.04	0.80
rs2043556 T>C (miR-605)	C		0.29/0.30	0.37	0.95	0.75	1.00	0.98	0.97	0.83
rs6505162 A>C (miR-423)	C		0.56/0.57	0.54	0.99	0.94	1.02	0.86	0.98	0.83
Intestinal-type Gastric Cancer										
rs4938723 T>C (miR-34b/c)	C		0.24/0.26	0.22	0.95	0.76	0.90	0.56	0.92	0.61
rs895819 T>C (miR-27a)	C		0.20/0.23	0.38	0.81	0.30	0.77	0.24	0.81	0.30
rs3746444 A>G (miR-499)	G		0.14/0.15	0.13	0.94	0.76	0.85	0.49	0.94	0.76
rs2043556 T>C (miR-605)	C		0.28/0.30	0.37	0.88	0.48	0.85	0.44	0.88	0.48
rs6505162 A>C (miR-423)	C		0.58/0.57	0.54	1.07	0.62	1.10	0.56	1.07	0.62
Diffuse-type Gastric Cancer										
rs4938723 T>C (miR-34b/c)	C		0.26/0.26	0.22	0.99	0.97	1.01	0.98	1.00	1.00
rs895819 T>C (miR-27a)	C		0.18/0.23	0.38	0.67	0.09	0.70	0.14	0.69	0.10
rs3746444 A>G (miR-499)	G		0.15/0.15	0.13	1.05	0.84	1.14	0.60	1.05	0.83
rs2043556 T>C (miR-605)	C		0.30/0.30	0.37	0.97	0.87	0.96	0.87	1.00	0.99
rs6505162 A>C (miR-423)	C		0.52/0.57	0.54	0.83	0.22	0.84	0.31	0.83	0.23
TNM I-II										
rs4938723 T>C (miR-34b/c)	C		0.29/0.26	0.22	1.22	0.27	1.16	0.44	1.20	0.31
rs895819 T>C (miR-27a)	C		0.21/0.23	0.38	0.84	0.46	0.86	0.52	0.85	0.46
rs3746444 A>G (miR-499)	G		0.13/0.15	0.13	0.82	0.43	0.79	0.37	0.83	0.46
rs2043556 T>C (miR-605)	C		0.32/0.30	0.37	1.18	0.42	1.17	0.48	1.19	0.38
rs6505162 A>C	C		0.58/0.57	0.54	1.09	0.61	1.09	0.64	1.05	0.76

(miR-423)										
TNM III-IV										
rs4938723 T>C (miR-34b/c)	C		0.23/0.26	0.22	0.88	0.42	0.86	0.40	0.86	0.33
rs895819 T>C (miR-27a)	C		0.20/0.23	0.38	0.80	0.27	0.80	0.32	0.82	0.31
rs3746444 A>G (miR-499)	G		0.16/0.15	0.13	1.14	0.52	1.26	0.32	1.14	0.53
rs2043556 T>C (miR-605)	C		0.29/0.30	0.37	0.95	0.76	0.99	0.95	0.95	0.80
rs6505162 A>C (miR-423)	C		0.56/0.57	0.54	0.97	0.81	0.97	0.84	0.96	0.74
H.pylori infected subjects										
rs4938723 T>C (miR-34b/c)	C		0.25/0.28	0.22	1.05	0.85	1.17	0.58	0.86	0.51
rs895819 T>C (miR-27a)	C		0.16/0.24	0.38	0.56	0.08	0.61	0.17	0.53	0.04
rs3746444 A>G (miR-499)	G		0.14/0.17	0.13	0.74	0.35	0.85	0.68	0.80	0.47
rs2043556 T>C (miR-605)	C		0.28/0.25	0.37	1.18	0.56	1.49	0.22	1.22	0.46
rs6505162 A>C (miR-423)	C		0.55/0.57	0.54	0.94	0.76	1.00	1.00	0.92	0.69
*H.pylori cag*PAI-positive subjects										
rs4938723 T>C (miR-34b/c)	C		0.25/0.23	0.22	1.24	0.49	1.59	0.19	1.10	0.75
rs895819 T>C (miR-27a)	C		0.16/0.20	0.38	0.64	0.31	0.77	0.60	0.66	0.31
rs3746444 A>G (miR-499)	G		0.15/0.17	0.13	0.77	0.52	0.79	0.61	0.86	0.70
rs2043556 T>C (miR-605)	C		0.30/0.28	0.37	1.25	0.52	1.60	0.24	1.12	0.73
rs6505162 A>C (miR-423)	C		0.56/0.61	0.54	0.78	0.35	0.88	0.66	0.84	0.48

1 Adjusted for sex, principal component (PC)1 and PC2. 2 Adjusted for age, sex, principal component (PC)1 and PC2. 3 Crude. AMR: Allele frequency in Ad Mixed Americans in 1000 genomes project, R2: R-square (a metric of quality of imputation), OR: Odds ratio, p-value< 0.01 is considered significant.
